# Insights into frequent asthma exacerbations from a primary care perspective and the implications of UK National Review of Asthma Deaths recommendations

**DOI:** 10.1038/s41533-018-0103-9

**Published:** 2018-09-19

**Authors:** Jieqiong Freda Yang, Rekha Chaudhuri, Neil C. Thomson, Nitish Ramparsad, Hugh O’Pray, Stephen Barclay, Sean MacBride-Stewart, Craig McCallum, Varun Sharma, Charles McSharry, Dianne Murray, Malcolm Shepherd, Wai-Ting Nicola Lee

**Affiliations:** 1 Asthma/COPD Clinical Research Centre, Gartnavel General Hospital, Glasgow & Institute of Infection, Immunity & Inflammation, University of Glasgow, Glasgow, UK; 2 Robertson Centre for Biostatistics, Institute of Health and Wellbeing, University of Glasgow, Glasgow, UK; 3 eHealth Strategy & Programmes, NHS Greater Glasgow & Clyde, Glasgow, UK; 4 Department of Gastroenterology, Glasgow Royal Infirmary, Glasgow, UK; 5 Pharmacy Prescribing Support Unit, NHS Greater Glasgow & Clyde, Glasgow, UK

## Abstract

The United Kingdom National Review of Asthma Deaths (NRAD) recommends that patients who require ≥3 courses of oral corticosteroids (OCS) for exacerbations in the past year or those on British Thoracic Society (BTS) Step 4/5 treatment must be referred to a specialist asthma service. The aim of the study was to identify the proportion of asthma patients in primary care that fulfil NRAD criteria for specialist referral and factors associated with frequent exacerbations. A total of 2639 adult asthma patients from 10 primary care practices in Glasgow, UK were retrospectively studied between 2014 and 2015. Frequent exacerbators and short-acting β_2_-agonist (SABA) over-users were identified if they received ≥2 confirmed OCS courses for asthma and ≥13 SABA inhalers in the past year, respectively. Community dispensing data were used to assess treatment adherence defined as taking ≥75% of prescribed inhaled corticosteroid (ICS) dose. The study population included 185 (7%) frequent exacerbators, 137 (5%) SABA over-users, and 319 (12%) patients on BTS Step 4/5 treatment. Among frequent exacerbators, 41% required BTS Step 4/5 treatment, 46% had suboptimal ICS adherence, 42% had not attended an asthma review in the past year and 42% had no previous input from a specialist asthma service. Older age, female gender, BTS Step 4/5, SABA over-use and co-existing COPD diagnosis increased the risk of frequent exacerbations independently. Fourteen per 100 asthma patients would fulfil the NRAD criteria for specialist referral. Better collaboration between primary and secondary care asthma services is needed to improve chronic asthma care.

## Asthma: A need for integrated care

An audit of asthma patients in Scotland reveals the need for better integration between primary care providers and secondary asthma specialists. The UK National Review of Asthma Deaths highlighted 19 recommendations to prevent avoidable asthma deaths, including specialist referrals for those who over-use reliever inhalers or are admitted to hospital regularly. Wai-Ting Lee at the University of Glasgow, Scotland, and co-workers conducted an audit of 2,639 adult asthma patients from across Glasgow between 2014 and 2015. Their data suggest around 15 per cent of active asthma sufferers would benefit from secondary specialist asthma referrals, yet 42 per cent of those with the most severe symptoms had not attended annual asthma reviews. The patients in this group were also more likely to expose themselves to avoidable asthma triggers such as smoking and over-use of reliever inhalers.

## Introduction

Asthma is the most prevalent chronic respiratory disease in the world affecting an estimated 358 million people in 2015.^1^ The prevalence of asthma is high in the United Kingdom compared to many other countries,^2^ ranging from 29.5% (18.5 million people) for lifetime symptoms suggestive of asthma to 5.7% (3.6 million people) for those with active, clinician-diagnosed-and-treated asthma.^3^ United Kingdom has the third highest death rates for asthma in high-income countries worldwide.^4^ Asthma generates a considerable workload in general practice, with some estimated 2.7 million GP consultations, 3.7 million nurse consultations and 54,000 out-of-hours calls for asthma in 2011–2012.^3^ The annual costs of asthma are estimated to be at least £1.1 billion with the majority incurred in primary care and ~10% in secondary care. There were 1370 asthma deaths across the United Kingdom in 2016, among them 133 in Scotland (data from National Records of Scotland) and 1237 in England and Wales (data collected from Office for National Statistics in England and Wales).

It is over 40 years since the first studies on asthma deaths in the United Kingdom were published in the 1970s.^5–7^ Despite the expansion of effective asthma treatment and development of evidence-based management guidelines such as those from British Thoracic Society (BTS)/Scottish Intercollegiate Guidelines Network (SIGN) and Global Initiative for Asthma (GINA),^4,8^ asthma continues to cause significant morbidity and unacceptably high mortality rates. Avoidable factors preceding deaths were repeatedly identified in UK regional investigations into asthma deaths.^9^ In 2014, the Royal College of Physicians UK published the National Review of Asthma Deaths (NRAD) report, ‘Why asthma still kills’.^10^ This was the first and largest national investigation of asthma deaths in the United Kingdom to establish the effectiveness of asthma management, identify potential avoidable factors and make recommendations to reduce the number of preventable deaths. NRAD requested information on 900 asthma deaths coded according to International Statistical Classification of Diseases and Related Health Problems 10th Revision between February 2012 and January 2013 in England, Wales and Scotland. A total of 740 patient records were returned and evaluated. After excluding 39% who were not asthma deaths, 276 possible asthma deaths were considered in detail by the confidential enquiry panels. One hundred ninety-five (70%) of those cases were confirmed as asthma deaths. The key findings were that the overall asthma management in primary and secondary care was considered good in less than one-fifth of those who died. Potentially avoidable factors were identified in two-thirds of cases. These findings led to 19 key recommendations related to the organisation of NHS services, medical and professional care, prescribing and medicines use, patient factors and perception of risk. These include patients must be referred to a specialist asthma service if they required ≥3 courses of systemic corticosteroids or ≥1 hospital admission in the previous 12 months or BTS Step 4/5 treatment to achieve control. These recommendations are similar to BTS/SIGN asthma guidelines, whereas GINA guidelines recommend referral in those who have ≥2 exacerbations per year or uncontrolled symptoms despite medium or high dose inhaled corticosteroids (ICS).^4,8^

The authors carried out an audit of asthma patients from ten primary care practices in the North Greater Glasgow area over a 12-month period between 2014 and 2015. The aims of the audit were first to identify asthma patients with recurrent exacerbations or on a high level of treatment who would merit referral to a secondary care asthma service, and second to study the biopsychosocial factors associated with frequent exacerbations.

## Results

### Study cohort

A total of 48,462 patients were registered with the ten participating primary care practices. A total of 4495 (9%) patients had an asthma diagnosis. A total of 2639 (5%) patients received at least one asthma reliever or preventer inhaler in the past 12 months and were included in the analysis as active asthma patients. Among those, 185 patients (7%) required ≥2 courses of oral corticosteroids (OCS) for asthma exacerbations in the past 12 months (frequent exacerbators). One hundred thirty-seven (5%) were prescribed ≥13 short-acting β_2_-agonist (SABA) inhalers in the past 12 months (SABA over-users). Three hundred nineteen (12%) were on BTS Step 4/5 treatment. A&E attendance or hospital admission was reported in 1.2% of patients. A total of 1724 (65%) had at least one of the recorded comorbidities including chronic obstructive pulmonary disease (COPD), diabetes, coronary heart disease, hypertension, eczema, rhinitis, gastro–oesophageal reflux disease, history of anxiety or depression and osteoporosis. Five hundred ninety-two (22%) were current smokers. Two hundred eighty-nine (11%) had a co-existing diagnosis of COPD. In this group, 22% were frequent exacerbators, 13% were SABA over-users and 43% were on BTS step 4/5 treatment. Characteristics of all active asthma patients are shown in Table 1. Characteristics of patients with co-existing asthma and COPD diagnoses are shown in Table 2. Characteristics of patients with asthma and no co-existing COPD diagnosis are shown in Table 3. Prevalence rates of frequent exacerbators and SABA over-users by practice are shown in Table 4.

**Table 1:** Characteristics of all active asthma patients

	All patients (*n* = 2639)	Non-exacerbators (*n* = 2454)	Exacerbators (*n* = 185)	*p* value	SABA ≤12 per year (*n* = 2502)	SABA over-users (*n* = 137)	*p* value
Age							
Mean ± SD	48.5 ± 17.4	47.8 ± 17.4	56.9 ± 16.1	*p* < 0.001	48.3 ± 17.5	51.7 ± 15.6	*p* = 0.016
Sex							
Female (%) Male (%)	1538 (58.3%) 1100 (41.7%)	1412 (57.6%) 1041 (42.4%)	126 (68.1%) 59 (31.9%)	*p* = 0.006	1464 (58.5%) 1037 (41.5%)	74 (54.0%) 63 (46.0%)	*p* = 0.339
Age of onset (years)							
<18 (%) ≥18 (%)	449 (17.0%) 2190 (83.0%)	432 (17.6%) 2022 (82.4%)	17 (9.2%) 168 (90.8%)	*p* = 0.005	423 (16.9%) 2079 (83.1%)	26 (19.0%) 111 (81.0%)	*p* = 0.609
Duration of asthma (years)							
Mean ± SD	19.3 ± 15.2	19.4 ± 15.3	19.2 ± 14.2	*p* = 0.866	19.2 ± 15.3	21.3 ± 12.4	*p* = 0.065
Smoking status							
Current smoker (%) Ex-smoker (%) Never smoked (%)	592 (22.4%) 699 (26.5%) 1348 (51.1%)	542 (22.1%) 635 (25.9%) 1277 (52.0%)	50 (27.0%) 64 (34.6%) 71 (38.4%)	*p* = 0.001	541 (21.6%) 650 (26.0%) 1311 (52.4%)	51 (37.2%) 49 (35.8%) 37 (27.0%)	*p* < 0.001
Comorbidities							
COPD (%) Diabetes (%) CHD (%) Hypertension (%) Eczema (%) GORD (%) Rhinitis (%) History of depression (%) History of anxiety (%) Osteoporosis (%)	289 (11.0%) 179 (6.8%) 176 (6.7%) 514 (19.5%) 253 (9.6%) 216 (8.2%) 476 (18.0%) 607 (23.0%) 265 (10.0%) 117 (4.4%)	225 (9.2%) 155 (6.3%) 152 (6.2%) 466 (19.0%) 243 (9.9%) 200 (8.1%) 442 (18.0%) 550 (22.4%) 247 (10.1%) 101 (4.1%)	64 (34.6%) 24 (13.0%) 24 (13.0%) 48 (25.9%) 10 (5.4%) 16 (8.6%) 34 (18.4%) 57 (30.8%) 18 (9.7%) 16 (8.6%)	*p* < 0.001 *p* = 0.001 *p* = 0.001 *p* = 0.026 *p* = 0.051 *p* = 0.781 *p* = 0.921 *p* = 0.011 *p* = 1.000 *p* = 0.008	253 (10.1%) 162 (6.5%) 165 (6.6%) 490 (19.6%) 234 (9.4%) 202 (8.1%) 450 (18.0%) 551 (22.0%) 246 (9.8%) 104 (4.2%)	36 (26.3%) 17 (12.4%) 11 (8.0%) 24 (17.5%) 19 (13.9%) 14 (10.2%) 26 (19.0%) 56 (40.9%) 19 (13.9%) 13 (9.5%)	*p* < 0.001 *p* = 0.013 *p* = 0.482 *p* = 0.657 *p* = 0.099 *p* = 0.339 *p* = 0.733 *p* < 0.001 *p* = 0.142 *p* = 0.008
Number of comorbidities							
0 (%) 1 (%) 2 (%) 3 (%) 4+ (%)	915 (34.7%) 875 (33.2%) 493 (18.7%) 233 (8.8%) 123 (4.7%)	886 (36.1%) 815 (33.2%) 434 (17.7%) 211 (8.6%) 108 (4.4%)	29 (15.7%) 60 (32.4%) 59 (31.9%) 22 (11.9%) 15 (8.1%)	*p* < 0.001	889 (35.5%) 835 (33.4%) 457 (18.3%) 210 (8.4%) 111 (4.4%)	26 (19.0%) 40 (29.2%) 36 (26.3%) 23 (16.8%) 12 (8.8%)	*p* < 0.001
BTS step at the end of study year							
1 (%) 2 (%) 3 (%) 4 (%) 5 (%) Unclear (%)	488 (18.5%) 749 (28.4%) 1056 (40.0%) 313 (11.9%) 6 (0.2%) 27 (1.0%)	483 (19.7%) 729 (29.7%) 972 (39.6%) 240 (9.8%) 3 (0.1%) 27 (1.1%)	5 (2.7%) 20 (10.8%) 84 (45.4%) 73 (39.5%) 3 (1.6%) 0 (0.0%)	*p* < 0.001	479 (19.1%) 716 (28.6%) 995 (39.8%) 284 (11.4%) 5 (0.2%) 23 (0.9%)	9 (6.6%) 33 (24.1%) 61 (44.5%) 29 (21.2%) 1 (0.7%) 4 (2.9%)	*p* < 0.001
ICS dose at the end of study year (BDP equivalent)							
Low (%) Medium (%) High (%) Not on ICS (%) Unclear (%)	979 (37.1%) 674 (25.5%) 316 (12.0%) 438 (16.6%) 232 (8.8%)	946 (38.5%) 612 (24.9%) 242 (9.9%) 433 (17.6%) 221 (9.0%)	33 (17.8%) 62 (33.5%) 74 (40.0%) 5 (2.7%) 11 (5.9%)	*p* < 0.001	937 (37.5%) 630 (25.2%) 286 (11.4%) 429 (17.1%) 220 (8.8%)	42 (30.7%) 44 (32.1%) 30 (21.9%) 9 (6.6%) 12 (8.8%)	*p* < 0.001
Asthma review in past year							
No (%) Yes (%)	1245 (47.2%) 1394 (52.8%)	1168 (47.6%) 1286 (52.4%)	77 (41.6%) 108 (58.4%)	*p* = 0.135	1185 (47.4%) 1317 (52.6%)	60 (43.8%) 77 (56.2%)	*p* = 0.468
Exacerbators							
No (%) Yes (%)	2454 (93.0%) 185 (7.0%)	—	—	—	2342 (93.6%) 160 (6.4%)	112 (81.8%) 25 (18.2%)	*p* < 0.001
SABA over-users							
No (%) Yes (%)	2502 (94.8%) 137 (5.2%)	2342 (95.4%) 112 (4.6%)	160 (86.5%) 25 (13.5%)	*p* < 0.001	—	—	—

Definitions: Frequent exacerbators, ≥2 courses of oral corticosteroids (OCS) for asthma exacerbations in the past 12 months; SABA over-users, prescribed ≥13 SABA inhalers in the past 12 months

*SD* standard deviation, *SABA* short-acting β2-antagonist, *COPD* chronic obstructive pulmonary disease, *CHD* coronary heart disease, *HBP* hypertension, *GORD* gastro–oesophageal reflux disease, *BTS* British Thoracic Society, *ICS* inhaled corticosteroids, *BDP* beclometasone diproprionate

**Table 2:** Characteristics of patients with co-existing asthma and COPD diagnoses

	All patients (*n* = 289)	Non-exacerbators (*n* = 225)	Exacerbators (*n* = 64)	*p* value	SABA ≤12 per year (*n* = 253)	SABA over-users (*n* = 36)	*p* value
Age							
Mean ± SD	66.2 ± 11.8	66.8 ± 12.0	63.9 ± 11.0	*p* = 0.065	66.8 ± 11.7	62.1 ± 12.1	*p* = 0.036
Sex							
Female (%) Male (%)	167 (57.8%) 122 (42.2%)	129 (57.3%) 96 (42.7%)	38 (59.4%) 26 (40.6%)	*p* = 0.882	145 (57.3%) 108 (42.7%)	22 (61.1%) 14 (38.9%)	*p* = 0.801
Age of onset (years)							
<18 (%) ≥18 (%)	13 (4.5%) 276 (95.5%)	10 (4.4%) 215 (95.6%)	3 (4.7%) 61 (95.3%)	*p* = 1.000	12 (4.7%) 241 (95.3%)	1 (2.8%) 35 (97.2%)	*p* = 0.918
Duration of asthma (years)							
Mean ± SD	17.9 ± 15.1	17.4 ± 15.7	19.9 ± 12.7	*p* = 0.197	17.7 ± 15.4	19.8 ± 13.0	*p* = 0.365
Smoking status							
Current smoker (%) Ex-smoker (%) Never smoked (%)	92 (31.8%) 157 (54.3%) 40 (13.8%)	70 (31.1%) 121 (53.8%) 34 (15.1%)	22 (34.4%) 36 (56.2%) 6 (9.4%)	*p* = 0.495	80 (31.6%) 137 (54.2%) 36 (14.2%)	12 (33.3%) 20 (55.6%) 4 (11.1%)	*p* = 0.877
Comorbidities							
Diabetes (%) CHD (%) Hypertension (%) Eczema (%) GORD (%) Rhinitis (%) History of depression (%) History of anxiety (%) Osteoporosis (%)	37 (12.8%) 58 (20.1%) 110 (38.1%) 7 (2.4%) 27 (9.3%) 29 (10.0%) 79 (27.3%) 37 (12.8%) 44 (15.2%)	27 (12.0%) 45 (20.0%) 93 (41.3%) 6 (2.7%) 22 (9.8%) 23 (10.2%) 59 (26.2%) 30 (13.3%) 36 (16.0%)	10 (15.6%) 13 (20.3%) 17 (26.6%) 1 (1.6%) 5 (7.8%) 6 (9.4%) 20 (31.2%) 7 (10.9%) 8 (12.5%)	*p* = 0.524 *p* = 1.000 *p* = 0.041 *p* = 1.000 *p* = 0.809 *p* = 1.000 *p* = 0.430 *p* = 0.832 *p* = 0.560	29 (11.5%) 52 (20.6%) 102 (40.3%) 5 (2.0%) 26 (10.3%) 23 (9.1%) 63 (24.9%) 29 (11.5%) 38 (15.0%)	8 (22.2%) 6 (16.7%) 8 (22.2%) 2 (5.6%) 1 (2.8%) 6 (16.7%) 16 (44.4%) 8 (22.2%) 6 (16.7%)	*p* = 0.104 *p* = 0.663 *p* = 0.043 *p* = 0.212 *p* = 0.221 *p* = 0.229 *p* = 0.017 *p* = 0.104 *p* = 0.805
Number of comorbidities							
0 (%) 1 (%) 2 (%) 3 (%) 4+ (%)	0 (0.0%) 66 (22.8%) 88 (30.4%) 84 (29.1%) 51 (17.6%)	0 (0.0%) 53 (23.6%) 64 (28.4%) 65 (28.9%) 43 (19.1%)	0 (0.0%) 13 (20.3%) 24 (37.5%) 19 (29.7%) 8 (12.5%)	*p* = 0.421	0 (0.0%) 61 (24.1%) 75 (29.6%) 73 (28.9%) 44 (17.4%)	0 (0.0%) 5 (13.9%) 13 (36.1%) 11 (30.6%) 7 (19.4%)	*p* = 0.576
BTS step at the end of study year							
1 (%) 2 (%) 3 (%) 4 (%) 5 (%) Unclear (%)	18 (6.2%) 26 (9.0%) 118 (40.8%) 123 (42.6%) 2 (0.7%) 2 (0.7%)	17 (7.6%) 24 (10.7%) 99 (44.0%) 82 (36.4%) 1 (0.4%) 2 (0.9%)	1 (1.6%) 2 (3.1%) 19 (29.7%) 41 (64.1%) 1 (1.6%) 0 (0.0%)	*p* = 0.002	17 (6.7%) 23 (9.1%) 105 (41.5%) 106 (41.9%) 1 (0.4%) 1 (0.4%)	1 (2.8%) 3 (8.3%) 13 (36.1%) 17 (47.2%) 1 (2.8%) 1 (2.8%)	*p* = 0.268
ICS dose at the end of study year (BDP equivalent)							
Low (%) Medium (%) High (%) Not on ICS (%) Unclear (%)	40 (13.8%) 95 (32.9%) 124 (42.9%) 16 (5.5%) 14 (4.8%)	38 (16.9%) 78 (34.7%) 82 (36.4%) 15 (6.7%) 12 (5.3%)	2 (3.1%) 17 (26.6%) 42 (65.6%) 1 (1.6%) 2 (3.1%)	*p* < 0.001	37 (14.6%) 87 (34.4%) 106 (41.9%) 15 (5.9%) 8 (3.2%)	3 (8.3%) 8 (22.2%) 18 (50.0%) 1 (2.8%) 6 (16.7%)	*p* = 0.004
Asthma review in past year							
No (%) Yes (%)	111 (38.4%) 178 (61.6%)	87 (38.7%) 138 (61.3%)	24 (37.5%) 40 (62.5%)	*p* = 0.981	100 (39.5%) 153 (60.5%)	11 (30.6%) 25 (69.4%)	*p* = 0.394
Exacerbators							
No (%) Yes (%)	225 (77.9%) 64 (22.1%)	—	—	—	204 (80.6%) 49 (19.4%)	21 (58.3%) 15 (41.7%)	*p* = 0.005
SABA over-users							
No (%) Yes (%)	253 (87.5%) 36 (12.5%)	204 (90.7%) 21 (9.3%)	49 (76.6%) 15 (23.4%)	*p* = 0.005	—	—	—

Definitions: Frequent exacerbators, ≥2 courses of oral corticosteroids (OCS) for asthma exacerbations in the past 12 months; SABA over-users, prescribed ≥13 SABA inhalers in the past 12 months

*SD* standard deviation, *SABA* short-acting β2-antagonist, *COPD* chronic obstructive pulmonary disease, *CHD* coronary heart disease, *HBP* hypertension, *GORD* gastro–oesophageal reflux disease, *BTS* British Thoracic Society, *ICS* inhaled corticosteroids, *BDP* beclometasone diproprionate

**Table 3:** Characteristics of patients with asthma and no co-existing COPD diagnosis

	All patients (*n* = 2350)	Non-exacerbators (*n* = 2229)	Exacerbators (*n* = 121)	*p* value	SABA ≤12 per year (*n* = 2249)	SABA over-users (*n* = 101)	*p* value
Age							
Mean ± SD	46.3 ± 16.7	45.9 ± 16.6	53.2 ± 17.1	*p* < 0.001	46.2 ± 16.8	47.9 ± 15.0	*p* = 0.268
Sex							
Female (%) Male (%)	1371 (58.4%) 978 (41.6%)	1283 (57.6%) 945 (42.4%)	88 (72.7%) 33 (27.3%)	*p* = 0.001	1319 (58.7%) 929 (41.3%)	52 (51.5%) 49 (48.5%)	*p* = 0.183
Age of onset (years)							
<18 (%) ≥18 (%)	436 (18.6%) 1914 (81.4%)	422 (18.9%) 1807 (81.1%)	14 (11.6%) 107 (88.4%)	*p* = 0.056	411 (18.3%) 1838 (81.7%)	25 (24.8%) 76 (75.2%)	*p* = 0.132
Duration of asthma (years)							
Mean ± SD	19.5 ± 15.2	19.6 ± 15.2	18.8 ± 15.0	*p* = 0.598	19.4 ± 15.3	21.8 ± 12.2	*p* = 0.059
Smoking status							
Current smoker (%) Ex-smoker (%) Never smoked (%)	500 (21.3%) 542 (23.1%) 1308 (55.7%)	472 (21.2%) 514 (23.1%) 1243 (55.8%)	28 (23.1%) 28 (23.1%) 65 (53.7%)	*p* = 0.863	461 (20.5%) 513 (22.8%) 1275 (56.7%)	39 (38.6%) 29 (28.7%) 33 (32.7%)	*p* < 0.001
Comorbidities							
Diabetes (%) CHD (%) Hypertension (%) Eczema (%) GORD (%) Rhinitis (%) History of depression (%) History of anxiety (%) Osteoporosis (%)	142 (6.0%) 118 (5.0%) 404 (17.2%) 246 (10.5%) 189 (8.0%) 447 (19.0%) 528 (22.5%) 228 (9.7%) 73 (3.1%)	128 (5.7%) 107 (4.8%) 373 (16.7%) 237 (10.6%) 178 (8.0%) 419 (18.8%) 491 (22.0%) 217 (9.7%) 65 (2.9%)	14 (11.6%) 11 (9.1%) 31 (25.6%) 9 (7.4%) 11 (9.1%) 28 (23.1%) 37 (30.6%) 11 (9.1%) 8 (6.6%)	*p* = 0.016 *p* = 0.050 *p* = 0.018 *p* = 0.358 *p* = 0.608 *p* = 0.235 *p* = 0.033 *p* = 1.000 *p* = 0.051	133 (5.9%) 113 (5.0%) 388 (17.3%) 229 (10.2%) 176 (7.8%) 427 (19.0%) 488 (21.7%) 217 (9.6%) 66 (2.9%)	9 (8.9%) 5 (5.0%) 16 (15.8%) 17 (16.8%) 13 (12.9%) 20 (19.8%) 40 (39.6%) 11 (10.9%) 7 (6.9%)	*p* = 0.201 *p* = 1.000 *p* = 0.789 *p* = 0.044 *p* = 0.088 *p* = 0.797 *p* < 0.001 *p* = 0.609 *p* = 0.035
Number of comorbidities							
0 (%) 1 (%) 2 (%) 3 (%) 4+ (%)	915 (38.9%) 809 (34.4%) 405 (17.2%) 149 (6.3%) 72 (3.1%)	886 (39.7%) 762 (34.2%) 370 (16.6%) 146 (6.6%) 65 (2.9%)	29 (24.0%) 47 (38.8%) 35 (28.9%) 3 (2.5%) 7 (5.8%)	*p* < 0.001	889 (39.5%) 774 (34.4%) 382 (17.0%) 137 (6.1%) 67 (3.0%)	26 (25.7%) 35 (34.7%) 23 (22.8%) 12 (11.9%) 5 (5.0%)	*p* = 0.012
BTS step at the end of study year							
1 (%) 2 (%) 3 (%) 4 (%) 5 (%) Unclear (%)	470 (20.0%) 723 (30.8%) 938 (39.9%) 190 (8.1%) 4 (0.2%) 25 (1.1%)	466 (20.9%) 705 (31.6%) 873 (39.2%) 158 (7.1%) 2 (0.1%) 25 (1.1%)	4 (3.3%) 18 (14.9%) 65 (53.7%) 32 (26.4%) 2 (1.7%) 0 (0.0%)	*p* < 0.001	462 (20.5%) 693 (30.8%) 890 (39.6%) 178 (7.9%) 4 (0.2%) 22 (1.0%)	8 (7.9%) 30 (29.7%) 48 (47.5%) 12 (11.9%) 0 (0.0%) 3 (3.0%)	*p* = 0.011
ICS dose at the end of study year (BDP equivalent)							
Low (%) Medium (%) High (%) Not on ICS (%) Unclear (%)	939 (40.0%) 579 (24.6%) 192 (8.2%) 422 (18.0%) 218 (9.3%)	908 (40.7%) 534 (24.0%) 160 (7.2%) 418 (18.8%) 209 (9.4%)	31 (25.6%) 45 (37.2%) 32 (26.4%) 4 (3.3%) 9 (7.4%)	*p* < 0.001	900 (40.0%) 543 (24.1%) 180 (8.0%) 414 (18.4%) 212 (9.4%)	39 (38.6%) 36 (35.6%) 12 (11.9%) 8 (7.9%) 6 (5.9%)	*p* = 0.007
Asthma review in past year							
No (%) Yes (%)	1134 (48.3%) 1216 (51.7%)	1081 (48.5%) 1148 (51.5%)	53 (43.8%) 68 (56.2%)	*p* = 0.361	1085 (48.2%) 1164 (51.8%)	49 (48.5%) 52 (51.5%)	*p* = 1.000
Exacerbators							
No (%) Yes (%)	2229 (94.9%) 121 (5.1%)	—	—	—	2138 (95.1%) 111 (4.9%)	91 (90.1%) 10 (9.9%)	*p* = 0.048
SABA over-users							
No (%) Yes (%)	2249 (95.7%) 101 (4.3%)	2138 (95.9%) 91 (4.1%)	111 (91.7%) 10 (8.3%)	*p* = 0.048	—	—	—

Definitions: Frequent exacerbators, ≥2 courses of oral corticosteroids (OCS) for asthma exacerbations in the past 12 months; SABA over-users, prescribed ≥13 SABA inhalers in the past 12 months

*SD* standard deviation, *SABA* short-acting β2-antagonist, *COPD* chronic obstructive pulmonary disease, *CHD* coronary heart disease, *HBP* hypertension, *GORD* gastro–oesophageal reflux disease, *BTS* British Thoracic Society, *ICS* inhaled corticosteroids, *BDP* beclometasone diproprionate

**Table 4:** Prevalence rates of frequent exacerbators and SABA over-users by practice

	Total number of registered patients	Active asthma patients, number (%)	Frequent exacerbators in each practice (%)	SABA over-users in each practice (%)	Patients living in 15% most deprived data zones in the SIMD ranking (%)
Practice 1	5450	251 (5)	4	3	10.2
Practice 2	11,574	583 (5)	4	2	5.5
Practice 3	3144	170 (5)	6	4	18
Practice 4	4644	214 (5)	7	2	10.6
Practice 5	6714	349 (5)	7	5	34.4
Practice 6	2478	136 (5)	7	10	20.5
Practice 7	2224	144 (6)	8	8	70
Practice 8	3088	225 (7)	8	9	71.8
Practice 9	5061	293 (6)	8	9	58
Practice 10	4085	274 (7)	14	5	36.9

*SABA* short-acting beta-2 agonist, *SIMD* Scottish Index of Multiple Deprivation

### Frequent exacerbators

Among 185 frequent exacerbators, 98 (53%) received two OCS prescriptions, 39 (21%) received three OCS prescriptions and 48 (26%) received four OCS prescriptions for asthma in the past 12 months. Compared to non-frequent exacerbators, they were older (mean age 57 ± 16 years vs 48 ± 17 years, *p* < 0.001), more likely to be female (68% vs 58%, *p* = 0.006) or ever-smokers (62% vs 48%, *p* = 0.001), and more likely to have adult-onset asthma (91% vs 82%, *p* = 0.005) (Table 1). A greater proportion had a co-existing diagnosis of COPD (35% vs 9%, *p* < 0.001). Other comorbidities including diabetes (13% vs 6%, *p* = 0.001), coronary heart disease (13% vs 6%, *p* = 0.001), hypertension (26% vs 19%, *p* = 0.026), history of depression (31% vs 22%, *p* = 0.011) and osteoporosis (9% vs 4%, *p* = 0.008) were more common. They were more likely to have at least one comorbidity (84% vs 64%, *p* < 0.001). A greater proportion of them were on BTS Step 4/5 treatment (41% vs 10%, *p* < 0.001) and were SABA over-users (14% vs 5%, *p* < 0.001). Three per cent of frequent exacerbators did not receive a prescription for ICS in the past 12 months. Forty-six per cent of those on ICS were taking <75% of prescribed ICS dose. Forty-two per cent had not attended an asthma review in primary care in the past 12 months. Forty-two per cent had no previous input from a specialist asthma service.

### SABA over-users

Among the 137 SABA over-users, 119 (86%) used between one and two SABA inhalers per month, 14 (10%) used between two and three SABA inhalers per month, and 4 (3%) used more than three SABA inhalers per month. Compared to patients using ≤12 SABA inhalers per year, they were older (mean age 52 ± 16 years vs 48 ± 16 years, *p* = 0.016) and more likely to be ever-smokers (73% vs 48%, *p* < 0.001) (Table 1). A greater proportion had a co-existing diagnosis of COPD (26% vs 10%, *p* < 0.001). Other comorbidities including diabetes (12% vs 7%, *p* = 0.013), history of depression (41% vs 22%, *p* < 0.001) and osteoporosis (10% vs 4%, *p* = 0.008) were more common. They were more likely to have at least one comorbidity (81% vs 64%, *p* < 0.001). A greater proportion of them were on BTS Step 4/5 treatment (22% vs 12%, *p* < 0.001) and were frequent exacerbators (18% vs 6%, *p* < 0.001). Seven per cent had not received a prescription for ICS in the past 12 months. Forty-four per cent had not attended an asthma review in primary care in the past 12 months.

### Predictors of asthma exacerbations

A multivariable logistic regression model was fitted to analyse the association between covariates and incident frequent exacerbations as a binary outcome. Independent predictors found to be significant were age, gender, BTS treatment step, SABA over-use and co-existing COPD diagnosis (Table 5). There was significant interaction between age and co-existing COPD diagnosis (*p* value for interaction = 0.029) (Table 6), with evidence for increased exacerbation risk in older patients without a co-existing COPD diagnosis, but no association between frequent exacerbations and age in patients with a co-existing COPD diagnosis. There was no evidence of interactions between COPD status and gender, BTS treatment step or SABA over-use.

**Table 5:** Logistic regression model for variables predicting frequent exacerbations (all patients)

	Full model estimates	Final model estimates
	Odds ratio (95% CI); *p* value	Odds ratio (95% CI); *p* value
Age (per 5 years)	1.08 (1.01, 1.14); *p* = 0.019	1.06 (1.01, 1.12); *p* = 0.020
Gender (male)	0.62 (0.43, 0.87); *p* = 0.006	0.64 (0.45, 0.89); *p* = 0.009
Asthma duration	1.00 (0.99, 1.01); *p* = 0.629	—
Ex-smoker Never smoked	0.81 (0.53, 1.24); *p* = 0.331 0.89 (0.58, 1.37); *p* = 0.606	—
Diabetes (yes)	1.62 (0.96, 2.72); *p* = 0.071	—
CHD (yes)	1.07 (0.63, 1.84); *p* = 0.794	—
Hypertension (yes)	0.76 (0.50, 1.14); *p* = 0.182	—
Eczema (yes)	0.75 (0.38, 1.50); *p* = 0.414	—
GORD (yes)	0.88 (0.50, 1.56); *p* = 0.664	—
Rhinitis (yes)	1.26 (0.82, 1.92); *p* = 0.285	—
Depression (yes)	1.15 (0.80, 1.65); *p* = 0.463	—
Anxiety (yes)	0.70 (0.40, 1.20); *p* = 0.190	—
Osteoporosis (yes)	0.87 (0.47, 1.61); *p* = 0.647	—
BTS step (1/2/unclear) BTS step (4/5)	0.43 (0.22, 0.82); *p* = 0.011 13.19 (1.70, 102.44); *p* = 0.014	0.29 (0.18, 0.46); *p* < 0.001 2.84 (1.97, 4.10); *p* < 0.001
ICS dose (medium) ICS dose (high) No ICS dose Unclear ICS dose	1.34 (0.76, 2.38); *p* = 0.313 0.26 (0.03, 2.20); *p* = 0.218 0.50 (0.19, 1.35); *p* = 0.172 0.99 (0.46, 2.10); *p* = 0.971	—
SABA over-use (yes)	2.20 (1.30, 3.71); *p* = 0.003	2.35 (1.42, 3.89); *p* = 0.001
COPD (yes)	2.06 (1.34, 3.18); *p* = 0.001	2.01 (1.34, 3.01); *p* = 0.001

*CHD* coronary heart disease, *GORD* gastro–oesophageal reflux disease, *BTS* British Thoracic Society, *ICS* inhaled corticosteroids, *SABA* short-acting β2-antagonist, *COPD* chronic obstructive pulmonary disease

**Table 6:** Logistic regression model for variables predicting frequent exacerbations (by co-existing COPD diagnosis status)

	With co-existing COPD	Without co-existing COPD	*p* value for interaction
	Odds ratio (95% CI); *p* value	Odds ratio (95% CI); *p* value	
Age (per 5 years)	0.94 (0.82, 1.06); *p* = 0.305	1.09 (1.03, 1.16); *p* = 0.003	0.029
Gender (male)	0.86 (0.47, 1.57); *p* = 0.629	0.56 (0.37, 0.85); *p* = 0.006	0.238
BTS step (1/2/unclear) BTS step (4/5)	0.37 (0.10, 1.33); *p* = 0.129 2.60 (1.39, 4.88); *p* = 0.003	0.29 (0.18, 0.48); *p* < 0.001 2.94 (1.87, 4.63); *p* < 0.001	0.8635
SABA over-use (yes)	2.75 (1.26, 6.02); *p* = 0.011	1.89 (0.94, 3.84); *p* = 0.076	0.485

*COPD* chronic obstructive pulmonary disease, *BTS* British Thoracic Society, *ICS* inhaled crticosteroids, *SABA* short-acting β2-antagonist

## Discussion

This is an observational study of asthma patients in an unselected primary care population using routine clinical data extracted from GP databases. It provided an estimate of the number of frequent exacerbators, SABA over-users and patients requiring BTS Step 4/5 treatment in a city population of patients having received at least one asthma reliever or preventer inhaler in the past 12 months. In the study population, 7% were frequent exacerbators, 5% were SABA over-users and 12% were on BTS Step 4/5 treatment. Fourteen per cent of primary care patients with active asthma would fulfil the NRAD and BTS/SIGN criteria for specialist asthma referral. Sixteen per cent would fulfil GINA criteria for specialist asthma referral. Among frequent exacerbators, there was a high prevalence of modifiable factors such as cigarette smoking, SABA over-use, treatment non-adherence and lack of regular asthma review. Multiple comorbidities were common. Older age, female gender, BTS Step 4/5, SABA over-use and co-existing COPD diagnosis increased the risk of frequent exacerbations independently.

The age, gender, smoking status, proportion of patients not requiring ICS and comorbidities of the study population were in general comparable to the demographic and clinical characteristics of the UK asthma population reported by several large studies using routine clinical data in primary care.^11–13^ There are differences where relevant data are available for comparison. The ethnicity of our study population was predominately white, whereas other ethnic groups were included in an analysis of primary care records in East London.^11^ The proportion of patients with difficult asthma (BTS Step 4/5 treatment) in our total study population was higher (12%) than reported from the East London study (~3%) and lower than reported from several UK national databases (~23%).^11,13,14^ It is unclear if differences between the cohorts reflect patient selection, differences in asthma service delivery, implementation of asthma guidelines, treatment adherence or disease severity. Nevertheless, we believe that the patients with asthma included in this analysis are likely to be comparable to most of the UK asthma population in primary care. The prevalence rate of frequent exacerbators in the study population of 7%, defined as ≥2 OCS courses for asthma in the past year, was comparable to rates reported in other large studies in the United Kingdom.^12,13^ Several risk factors known to be associated with frequent exacerbations in primary care^12,14^, including older age, female gender, cigarette smoking, comorbidities and treatment non-adherence, were more common in frequent exacerbators compared to non-exacerbators in our asthma population.

Cigarette smoking in asthma is associated with worse clinical outcomes.^15^ The 2016 Scottish Health Survey reported that one in five adults (21%) in the general population were current cigarette smokers^16^, a rate similar to the study population, although lower than that in frequent exacerbators (27%). National smoking cessation data published by Information Services Division Scotland showed that only 7% of smokers used the NHS smoking cessation services across Scotland in 2015/16.^17^ The self-reported quit rate was 37% at 1 month and 22% at 3 months. Respiratory diagnosis is not captured in the database, so it is not known if the usage of smoking cessation services and quit rates in asthmatics differ from the general smoking population in Scotland. To optimise smoking cessation intervention in asthmatic smokers, more data on the smoking pattern, current uptake of NHS smoking cessation services, quit rates and obstacles to quitting are needed, especially in those with poorly uncontrolled symptoms and frequent exacerbations. Smoking cessation remains a top priority in this group.

ICS non-adherence is common and a major cause of poor asthma outcomes. One study estimated 24% of asthma exacerbations were attributable to ICS non-adherence and exacerbation rates were significantly lower in patients taking ≥75% of prescribed ICS dose.^18^ Only 54% of frequent exacerbators were taking ≥75% of prescribed ICS dose in our study. Repeated instruction on inhaler technique and self-management education has been shown to improve adherence.^19,20^ Using objective adherence measures such as prescription refill records or electronic dose monitoring would allow a more meaningful concordance discussion between clinicians and patients. Common factors of ICS non-adherence such as dissatisfaction with the inhaler device, complex inhaler regime, fear of side effects and lack of instruction are amenable to change.^21^ Medicine costs are not a factor in treatment non-adherence in Scotland as prescriptions are free, unlike other parts of the United Kingdom where patients from low socio-economic backgrounds may be disadvantaged. Involvement of community pharmacists to deliver inhaler technique checks, identify inappropriate use of inhalers and promote smoking cessation would help to capture patients at the point of use. Overuse of SABA inhalers is associated with poor asthma control,^22^ increased risk of exacerbations,^23,24^ hospitalisation^11^ and death from asthma.^10,25^ Only 18% of SABA over-users were frequent exacerbators. This suggests that a majority of SABA over-users had persistent symptoms but were not prone to exacerbations. Their excessive SABA use could be related to psychological dependence on SABA inhalers, dysfunctional breathing or psychological distress.^26,27^ Patients with a co-existing COPD were more likely to over-use SABA inhalers (12% vs 4% for patients without a co-existing COPD), and this may reflect their need for more bronchodilator therapy. It is not known how many SABA over-users were on a long-acting anti-muscarinic inhaler. Seven per cent of SABA over-users were not on ICS and 31% were on low-dose ICS. This suggests that these patients might not be on adequate preventer medication. They might not have a personalised action plan or fully understand the rationale of asthma medication leading to over-reliance on SABA inhalers to control symptoms. Two key recommendations from NRAD were to improve patient engagement with regular asthma review, in order to reinforce the appropriate use of inhalers and self-management; and to use electronic surveillance of prescribing to alert clinicians to inappropriate reliever and preventer prescribing. Patients who over-order SABA prescriptions and under-order ICS prescriptions are most at risk of adverse outcomes and should be specifically targeted for asthma review.

In this study, a majority of frequent exacerbators presented with asthma symptoms after the age of 18 years. Previous studies suggest that adult-onset asthma has distinct phenotypic characteristics that are more related to environmental risk factors such as cigarette smoking than early-onset asthma.^28^ NRAD data suggest that adult-onset asthma may be a risk factor for asthma deaths; 69% of deaths, for whom information was available, occurred in patients diagnosed after the age of 15 years.^10^ More research is needed to improve the understanding of the natural history of adult-onset asthma and its pathophysiology in order to better target new treatment and improve outcomes.

The commonly encountered problem of diagnostic uncertainty in the management of asthma was highlighted by the number of miscoded asthma deaths in the NRAD report. Out of 276 possible asthma deaths, the confidential enquiry panels concluded that 195 (71%) people died from asthma but 81 people did not have asthma, had asthma but did not die from asthma, or there was not enough information to make a decision. Out of the 195 cases that had confirmation of asthma deaths, 42 (22%) were considered by their own clinicians to have COPD and asthma. Some asthma deaths may have been missed (not included in the 195 cases) because they were coded as COPD deaths. In addition, there was no documented rationale for the diagnosis of asthma in 64 (33%) of the 195 cases. One hundred (51%) of the 195 patients who died were diagnosed on the basis of recurrent symptoms, 34 (17%) on physiological measurement of lung function and 66 (34%) on the response to asthma medication. In our study, we did not look into the rationale for asthma diagnosis except in patients who had attended the secondary care asthma clinic, so some patients might have been misdiagnosed. For patients with co-existing asthma and COPD diagnoses, it is unclear how many of them truly had overlapping asthma and COPD or were simply miscoded when one diagnosis replaced another without updating the medical records. It is likely that some patients were miscoded. This is a major limitation of using routine primary care data based on disease coding. Clarification of diagnosis is one of the main indications for referral to a specialist asthma service, universally recommended by the NRAD report and other asthma guidelines.

A proportion of patients with asthma symptoms uncontrolled by standard doses of asthma medication would have other factors contributing to persistent symptoms. Failure to recognise and modify these factors would result in inappropriate treatment escalation and adverse outcomes as highlighted in the NRAD report. One proposed strategy to improving the management of asthma in primary care is SIMPLES.^29^ It provides a structured approach to evaluate factors that influence asthma control including smoking status (S), inhaler technique (I), monitoring (M), pharmacotherapy (P), lifestyle (L), education (E) and support (S). Patients who remain poorly controlled after applying this structured review should then be referred to a specialist asthma service for further diagnostic evaluation and consideration of treatment escalation. Both NRAD and BTS/SIGN guidelines recommend that patients should be followed up within 48 h of each attendance for an acute asthma attack. This would reduce the likelihood of patients re-attending for more treatment when treatment runs out before the original attack is over. Moreover, there is a need for closer collaboration between primary and secondary care clinicians. An integrated asthma care model was tested in the Grampian Asthma Study of Integrated Care in the 1990s, in which chest physicians reviewed patients in outpatient clinic annually and interim reviews took place in general practice.^30^ Through a computer patient record system, patients entered information about their symptoms, OCS courses and hospital admissions. The GP entered information about SABA use, OCS prescriptions and medication changes. The consultant would review the records and suggest changes to treatment if necessary. The study showed moderately severe asthma patients had similar clinical outcomes compared to the conventional group. Patients who had integrated care did benefit financially and considered themselves to have a higher level of asthma control compared to those who received conventional care. This model of integrated care may be the way forward.

### Strengths and limitations

There is currently no direct link between community dispensing data to individual patient medical records stored in primary or secondary care in the United Kingdom. Other UK studies had used prescription data based on electronic prescriptions issued by GPs to assess medication usage and treatment adherence.^11–14^ In this study, we used real life dispensing data submitted by community pharmacies for administrative purpose and linked them to individual patients by their Community Health Index (CHI) number, a unique 10-digit numeric identifier allocated to each patient on their first registration with the health service in Scotland. This would provide a more accurate evaluation of treatment adherence by excluding non-dispensed prescriptions. A major strength of this study is that each OCS prescription in frequent exacerbators was manually checked against clinical records to confirm that it was prescribed for an asthma exacerbation. This study has a number of limitations. Data on body mass index were lacking, and as a result, we were not able to explore the relationship between obesity and frequent exacerbations. It is well recognised that obese asthmatics respond less well to treatment, and this may have a negative impact on treatment adherence.^31^ There was no record of whether an invitation letter to asthma review had been sent or if patients had not attended appointments made. The number of patients issued with a personalised asthma action plan is not known.

The proportion of active asthma patients in a general primary care population who would warrant specialist asthma referral was estimated to be 14–16%. A high prevalence of modifiable factors in patients with frequent exacerbations was identified. These include cigarette smoking, SABA over-use, treatment non-adherence and lack of regular asthma review, consistent with the findings in the NRAD report. Independent predictors for frequent exacerbations were found to age, gender, BTS treatment step, SABA over-use and co-existing COPD diagnosis. There was significant interaction between age and co-existing COPD diagnosis, with evidence for increased exacerbation risk in older patients without a co-existing COPD diagnosis, but no association between frequent exacerbations and age in patients with a co-existing COPD diagnosis. It is clear that primary and secondary specialist asthma services need to work more closely together to provide integrated asthma care, in order to reduce exacerbations and prevent asthma deaths. This could be achieved by employing a structured approach to managing asthma patients in primary care and referring those with diagnostic uncertainty or therapy-resistant asthma to a specialist asthma service.

## Methods

### Study cohort

Ten GP practices in North Glasgow were invited to participate in this retrospective study of patients diagnosed with asthma in each practice between 2014 and 2015. The study cohort was identified by searching primary care EMIS-web databases for patients who had been coded with a diagnosis of asthma using computer software by an IT specialist. The process of how the study cohort was established is outlined in supplementary information on 'Data Extraction from GP databases'. Data on demographics (age, gender, age of onset and duration of asthma), smoking history, comorbidities (COPD, diabetes, coronary heart disease, hypertension, eczema, rhinitis, gastro–oesophageal reflux disease, history of anxiety or depression and osteoporosis), inhaled therapy (BTS Step) and number of OCS prescriptions were collected in patients aged ≥18 years and those who received at least one asthma reliever or preventer inhaler prescription in the past 12 months. Permission was obtained from individual GP practices to identify patients from their practice who had episodes of A&E attendance or hospital admission for asthma during the study period and review related clinical information from secondary care. Permission was granted from management of NHS Greater Glasgow & Clyde Health Board to access this data. Acute Information Team of the NHS Greater Glasgow & Clyde Health Board generated a list of patient episodes covering the study period for each practice. Each patient was identified by their CHI number. Each acute episode was identified by the date of admission and date of discharge. The causes of admissions and A&E attendances were checked against individual secondary care records. Patients were identified as a frequent exacerbator if they had ≥2 acute OCS courses for asthma exacerbations in the past 12 months. Prescription data of each frequent exacerbator were individually examined to exclude OCS prescribed for alternative diagnoses. Two courses of OCS prescribed within 1 week of each other were considered as one course of treatment. Community dispensing data were extracted from NHS central dispensing administrative data to assess treatment adherence in frequent exacerbators. The number of ICS prescriptions collected over a 12-month period between 2014 and 2015 was counted to calculate the total ICS dose (beclometasone diproprionate equivalent) received during this period. The average daily ICS dose received was then calculated. Treatment adherence was defined as taking ≥75% of prescribed ICS dose. Patients who used ≥13 SABA inhalers in the past 12 months were grouped as SABA over-users. All data were anonymised and managed in compliance with NHS Caldicott Guardian principles.

### Statistical analysis

Continuous data were summarised as mean ± standard deviation and compared using unpaired Student’s *t* test. Categorical data were summarised as number (%) and compared by Fisher’s exact test. A multivariable logistic regression model was fitted to further analyse the association between covariates and frequent exacerbations as a binary outcome. All covariates were entered into a manual backward selection procedure, in which covariates were sequentially removed if they failed to provide an improvement in model fit at a threshold of *p* < 0.05; at each step the covariate with the largest *p* value was removed. Covariates significant in predicting frequent exacerbations were age, gender, BTS treatment step, SABA over-use and co-existing COPD diagnosis. Both the full model and that of the final model odds ratio, 95% confidence interval (CI) and *p* values are shown in Table 5. The final model was then extended to include all two-way interactions with co-existing COPD diagnosis. Odds ratios for frequent exacerbations were estimated for those with and without a co-existing COPD diagnosis and are reported with 95% CI, *p* values, along with *p* value for each interaction term in this model (Table 6). All analyses were performed using the statistical software R version 3.1.2. (ref. 32)

## Electronic supplementary material

Data extraction from GP databases

## Data Availability

Data are available on application to the corresponding author.

## Electronic supplementary material

**Supplementary information** accompanies the paper on the *npj Primary Care Respiratory Medicine* website (10.1038/s41533-018-0103-9).
