# Supplementary material for: Insights into frequent asthma exacerbations from a primary care perspective and the implications of UK National Review of Asthma Deaths recommendations
Source: NPJ Prim Care Respir Med. 2018 Sep 19;28:35. doi: 10.1038/s41533-018-0103-9 (PMC6145932; doi:10.1038/s41533-018-0103-9)
Supplement: Supplementary file 1 — Data extraction from GP databases [file 41533_2018_103_MOESM1_ESM.pdf]

Data extraction from GP databases

All practices involved with the audit use EMIS PCS as their core clinical system.

Data was extracted from EMIS PCS using the following tools:

- Escro – a data collection tool produced by Albasoft to extract data from GP clinical systems in Scotland.
- EMIS PCS Population Manager – the EMIS reporting module used to generate QoF figures.
- EMIS Web Streaming – An additional EMIS product which provides enhanced reporting functionality beyond the base EMIS PCS system.

MS Excel was used to collate and summarise the results of the data extracted. The following table lists each of the worksheets used in the summarisation process. The source of the data and criteria for each query are listed. Unless otherwise noted each extract excludes patients under the age of 18. Where possible the sub-queries limited data to patients coded with Asthma, however to reduce the complexity of the queries this wasn't always practical.

The summarised data was transferred to a separate final workbook with the underlying formulas removed. The following table indicates which worksheets were transferred to the final data workbook.

Sheet	Source	Criteria	Notes
Coded Asthma – Unique List	ESCRO	H33..%, H3120, 173A	This worksheet holds all patients aged 18 and over who were ever coded with Asthma. This forms the master list of patients, with each patient listed only once. The results of the other queries are summarised on this worksheet.<br><br>The content of this worksheet is transferred to the final data workbook with the formulas removed.<br><br>The summarised data is also replicated on the Prednisolone worksheet below for convenience.

All Asthma Coding	ESCRO	H33..%, H3120, 173A	Lists all the individual Asthma codes for each patient in date ascending order. This is used to derive earliest diagnosis date.
Prednisolone	ESCRO	Drug name Like 'Prednisolone%' And Like '%tablet%'<br><br>Date of script issue up to 12 months prior to search run date.	Gives a list of every Prednisolone script (tablets only) for the Asthma patients during the 12 months prior to the search run date.<br><br>Asthma patients only extracted.<br><br>This worksheet is transferred to the final data workbook and contains the same summarised data as the 'Coded Asthma – Unique List' worksheet.
COPD	ESCRO	H3..., H31..%, H32..%, H36.., H37.., H38..<br>H39.., H3A.., H3y.., H3z.., H5832	Simplified QoF diagnosis code list. May include some non-QoF patients.
Diabetes	ESCRO	C10.., C109J, C109K, C10C., C10D., C10E.%, C10F.%, C10G.%, C10H.%, C10M.%, C10N.%, C10P.%, PKyP.	Simplified QoF diagnosis code list. May include some non-QoF patients.
CHD	ESCRO	G3...%	Simplified QoF diagnosis code list. May include some non-QoF patients.<br><br>Asthma patients only extracted.
Hypertension	ESCRO	G2..., G20..%, G24..%, G25..%<br>G26…, G28…, G2y.., G2z…<br>Gyu2., Gyu20	Simplified QoF diagnosis code list. May include some non-QoF patients.
Eczema	ESCRO	M11..%	Asthma patients only extracted.
GORD (or reflux)	ESCRO	J10y4 or 1955.	Asthma patients only extracted.
Rhinitis or Sinusitis	ESCRO	H120.%, H17..%, H13..%	Asthma patients only extracted.

Depression	ESCRO	E0013, E0021, E112.%, E113.%<br>E118., E11y2, E11z2, E130.<br>E135., E2003, E291., E2B..<br>E2B1., Eu204, Eu251, Eu32.%<br>Eu33.%, Eu341	Simplified QoF diagnosis code list. May include some non-QoF patients.<br><br>The presence of a code in the record is not an indication that this is a current problem for the patient. The date of latest code is added to the summary sheet for information.
Anxiety	ESCRO	E200.	The presence of a code in the record is not an indication that this is a current problem for the patient. The date of latest code is added to the summary sheet for information.<br><br>Asthma patients only extracted
Osteoporosis	ESCRO	N330.%, N3313, N3316, N3318, N3319<br>N331A, N331B, N331H, N331J, N331K<br>N331L, N331M, NyuB0, NyuB1, NyuB8<br>N3314, N3315, N3746, NyuB2	Simplified QoF diagnosis code list. May include some non-QoF patients.
Learning Difficulties	ESCRO	E3...%, Eu7..%, Eu814, Eu815, Eu816, Eu817<br>Eu81z, Eu818, 918e.	Simplified QoF diagnosis code list. May include some non-QoF patients.
Ethnicity	ESCRO	9i%, 9S%	Asthma patients only extracted.<br><br>Added a lookup table to include the description on summary sheet.
Smoking Status	ESCRO	137%	Simplified QoF coding – this will include some codes which don't give a true smoking status.<br><br>Codes are listed in date descending order, with the latest code included on the summary sheet.<br><br>Asthma patients only extracted.<br><br>Added a lookup table to include the description on summary sheet.

Asthma Review	ESCRO	66YJ., 66YK., 66YQ.<br>66YR., 8B3j., 9OJA.	Codes listed in date descending order. The latest date is included on the summary.
Pop Man Asthma Reg	EMIS PCS	QoF Asthma Register	This list is taken from EMIS PCS Population Manager and manually copied to this sheet.
Asthma Treatment	EMIS Web	BNF Chapters 3.1, 3.2 or 3.3<br><br>Date of script issue up to 12 months prior to search run date.	During testing it was found that the ESCRO extract tool was not giving full data for queries based on BNF chapter.<br><br>Data from an EMIS Web query is manually copied into the worksheet.<br><br>Asthma patients only extracted.
Treatment Detail	EMIS Web	BNF Chapters 3.1, 3.2 or 3.3<br><br>Date of script issue up to 12 months prior to search run date.	This is the same criteria as the Asthma Treatment query but lists the individual medication for each patient.<br><br>This data is copied into the final data workbook.

Problem List	EMIS Web	EMIS Problem Status = Significant and Active	This extract is an attempt to give an indication of how many significant conditions other than Asthma the patient has.<br><br>Coding in EMIS is made more prominent in the record by flagging it as a significant problem. Some practices use this status for operations and important administrative coding.<br><br>Once the data is copied into excel further processing is done in an attempt to ignore 'non-diagnosis' coding before a count of these codes is added to the summary sheet.<br><br>Codes beginning A to S are counted as these are usually diagnosis codes in the Read coding hierarchy. Codes beginning 1Z1 are also counted as these are the QoF CKD codes. Further criteria is added to ignore codes beginning H33 (to avoid double counting Asthma), and codes beginning 'PCS' or 'EMI' (these are not official Read codes).<br><br>The final number reported should be viewed as a broad indication as the quality of data recorded can vary between practices – duplicate codes can be common.

Notation: The % symbol is used as a wildcard. For Read code searches the notation H33..% would indicate code H33.. and all sub-codes in the Read code hierarchy are selected.
